# Evaluation of a novel quantitative canine species-specific point-of-care assay for C-reactive protein

**DOI:** 10.1186/s12917-018-1415-2

**Published:** 2018-03-20

**Authors:** Sarah Hindenberg, Melanie Keßler, Sabine Zielinsky, Judith Langenstein, Andreas Moritz, Natali Bauer

**Affiliations:** 1 Department of Veterinary Clinical Sciences, Clinical Pathology and Clinical Pathophysiology, Justus-Liebig-University Giessen, Frankfurter Str. 126, 35392 Giessen, Germany; 2 SYNLAB Vet GmbH, Augsburg, Germany

**Keywords:** Acute phase protein, C-reactive protein, Canine, Method validation, Point-of-care assay, Point-of-care analyzer, Total allowable error, Bias, Heparin plasma, Interference

## Abstract

**Background:**

Species-specific point-of-care tests (POCT) permit a rapid analysis of canine C-reactive protein (CRP), enabling veterinarians to include CRP in clinical decisions. Aim of the study was to evaluate a novel POCT for canine CRP (Point Strip™ Canine CRP Assay) run on a small in-house-analyzer (Point Reader™ V) using lithium heparin plasma and to compare assay performance to an already established canine CRP assay (Gentian Canine CRP Immunoassay) run on two different bench top analyzers serving as reference methods (ABX Pentra 400, AU 5800).

Linearity was assessed by stepwise dilution of plasma samples with high CRP concentrations. Limit of quantification (LoQ) was determined by repeated measurements of samples with low CRP concentrations. Coefficient of variation (CV) at low (10–50 mg/l), moderate (50–100 mg/l), and high (100–200 mg/l) CRP concentrations was investigated as well as possible interferences. Method comparison study was performed using 45 samples of healthy and diseased dogs. Quality criteria were fulfilled if the total observed error (TE_obs_ = 2CV% + bias%) was below the minimal total allowable error of 44.4% (TE _min_). Additionally, a reference range (*n* = 60 healthy dogs) was established.

**Results:**

Linearity was present at CRP concentrations of 10–132 mg/l (≙ 361 mg/l CRP with reference method) with a LoQ set at 10 mg/l. At moderate to high CRP concentrations, intra- and inter-assay CVs were ≤ 8% and ≤ 11% respectively, while CVs ≤ 22% and ≤ 28% were present at low concentrations. No interferences were observed at concentrations of 4 g/l hemoglobin, 800 mg/l bilirubin and 8 g/l triglycerides. Method comparison study demonstrated an excellent correlation with both reference methods (*r* = 0.98 for ABX Pentra 400; 0.99 for AU 5800), though revealing a proportional bias of 19.7% (ABX Pentra 400) and 10.7% (AU 5800) respectively. TE_obs_ was 26.7–31.9% and 16.7–21.9% and thus < TE_min_. Healthy dogs presented with CRP values ≤11.9 mg/l.

**Conclusions:**

The POCT precisely detects canine CRP at clinically relevant moderate and high CRP concentrations. The assay correlates well with both reference methods. Due to the bias, however, follow-up examinations should be performed with the same assay and analyzer.

## Background

C-reactive protein (CRP) is an important major acute phase protein (APP) in the dog. APPs are an integral part of innate immune response and change their serum concentration in adaption to a systemic inflammation [1–4]. Contrary to classic markers of inflammation as the white blood cell count, APPs react more rapidly and with a shorter half-life period [1, 5]. According to their kinetics, APPs are classified as slowly and mildly reacting minor and moderate APP or as rapidly reacting major APP, which increase 100- to 1000-fold within 24-48 h and decrease rapidly after disappearance of the inflammatory stimulus [3]. These sensitive markers of inflammation have been shown to increase in response to infectious diseases [6–9], immune mediated diseases [10–12], neoplasia [10, 13, 14], and surgery [15].

However, in the past, the measurement of canine CRP was hampered by the lack of a species-specific test available for veterinary practices and clinics.

Different assays for canine CRP were developed or adopted from human medicine and there was a shift from heterologous to homologous immunoassays which provide more reliable results [16–19]. Recently, a commercially available canine CRP assay was introduced on the market that is designed to be run on automated large bench top analyzers. First evaluations demonstrated its capability to detect canine CRP with a high precision and accuracy [20]. However, patients with severe inflammatory processes are often presented as emergency cases, so that a rapid measurement of CRP is desirable.

In human medicine, near-patient assays are available in order to fulfill this aim [21, 22].

Few canine species-specific CRP in-house tests have been evaluated in the past. The first point-of-care tests (POCT) were only semiquantitative, providing results of limited benefit and leading sometimes to falsely positive results [23–26]. Later, quantitative assays became available [27, 28]. Method comparison studies have often been performed with a manual ELISA serving as reference method, which, however, is impaired by a lack of precision.

Recently, a novel quantitative homologous bedside canine CRP assay (Point Strip™ Canine CRP Kit, USHIO Europe B.V., BC Oude Meer, The Netherlands) run on the in-house analyzer Point Reader™ V, USHIO Europe B.V., BC Oude Meer, The Netherlands became commercially available. The Point Strip™ Canine CRP assay for the Point Reader™ V is a test strip based colloidal gold immunochromatographic assay with species-specific rabbit anti-dog-CRP antibodies, which form complexes with canine CRP. These complexes migrate by capillary action through the membrane and are bound to the test line. Remaining non-bound antibodies are bound to the control line. The same test principle was used in a semiquantitative assay before [25].

Thus, it was the aim of our study to evaluate the ease of use and the test performance including linearity and lower limit of quantification (LoQ), precision and total observed error (TE_obs_) as well as interferences with bilirubin, hemoglobin and lipid of the canine species-specific in-house CRP assay run on the Point Reader™ V. In a method comparison study, the assay was compared with a canine CRP test which has been validated before [20, 29] run on two automated analyzers serving as reference methods. Furthermore, the reference range was evaluated. The hypothesis was that the investigated point-of-care analyzer is easy-to-use and that the results obtained with the POCT correlate well with the reference bench top analyzers.

## Methods

The prospective study was performed between September 2016 and July 2017.

### Measurement of CRP

Analyses were performed on surplus lithium heparin plasma submitted for CRP analysis to the Department of Veterinary Clinical Sciences, Clinical Pathology and Clinical Pathophysiology, Justus-Liebig-University Giessen, Germany. Samples were analyzed immediately (< 1 h after blood collection) with the Gentian Canine CRP Immunoassay (Gentian AS, Moss, Norway), which served as the reference assay, on the ABX Pentra 400 clinical chemistry analyzer (ABX Horiba, Montpellier, France) as described before [29]. The initially performed CRP analysis as part of routine diagnostics was used to assign the sample to one of the three concentration ranges: CRP 10–50 mg/l (low); 50–100 mg/l (medium), and 100–200 mg/l (high). The application of reagents and samples and the measurements on the in-house analyzer as well as on the large bench top analyzers were done by a single trained person for each analyzer according to the manufacturer’s instructions. In the method comparison study, the Point Strip™ Canine CRP POCT run on the Point Reader™ V (test and analyzer combination is subsequently just named “Point Reader™ V” or “POCT”) was compared to the reference assay run on two bench top analyzers (ABX Pentra 400, ABX Horiba, Montpellier, France and AU 5800, Beckman Coulter, Krefeld, Germany).

#### Method validation

##### Linearity and recovery

Linearity was evaluated by manual stepwise dilution of two canine lithium heparin plasma samples with markedly increased CRP concentration of ~ 245 and 360 mg/l determined on the ABX Pentra 400 analyzer which was used as a reference method. Serial dilution resulted in specimens with 1.0, 0.8, 0.6, 0.4, 0.2, 0.1, 0.05, and 0.025 of the original CRP concentration. All diluted aliquots were analyzed in triplicates in a single run. For all dilution steps % recovery rate was evaluated by comparison of expected and measured results. Furthermore, the dilution series was used for calculation of the recovery rate. The experiment was conducted twice to verify the measurements.

##### Precision and lower limit of quantification

Precision was assessed at three CRP concentration levels, i.e. low, moderate and high CRP concentrations. Intra- and inter-assay coefficients of variation (CVs) for the POCT were calculated from replicate measurements performed with samples of eight dogs each, whereby four samples of the lowest concentration range and two samples each of the moderate and high CRP concentration range were included. For assessment of intra-assay CV, ten replicate measurements were performed (*n* = 8 dogs). Inter-assay CV was calculated from single measurements performed on seven consecutive days (*n* = 8 dogs).

For assessment of LoQ, three lithium heparin plasma samples with CRP concentrations close to zero (20 mg/l, 10 mg/l, 0.8 mg/l) were assessed 20 times in a single run without recalibration as previously recommended [30].

##### Interferences

In order to investigate possible interferences, aliquots of a canine lithium heparin plasma sample with a medium concentration of ~ 50 mg/l CRP as assessed on the ABX Pentra 400 were spiked with 800 mg/l bilirubin (Bilirubin - ≥98%, powder, Sigma-Aldrich Co. LLC., St. Louis, Missouri, USA), 4 g/l hemoglobin (hemoglobin from bovine blood, lyophilized powder, Sigma-Aldrich Co. LLC., St. Louis, Missouri, USA) or 8 g/l 20% soy bean emulsion (Intralipid 20%, Fresenius Kabi Canada, Ontario, Canada). For assessment of the effect of hyperbilirubinemia, 20 mg bilirubin was dissolved in 1 ml of 0.1 M NaOH obtaining a stock solution of 20 g/l. Subsequently, 5 μl of the product was added to 120 μl of a non-spiked lithium heparin plasma sample to achieve a bilirubin level of 800 mg/l.

A stock solution containing 100 g/l hemoglobin was prepared by diluting 30 mg lyophilized bovine hemoglobin in 0.3 ml 0.09% NaCl. Then, 5 μl of the solution was added to 120 μl non-spiked lithium heparin plasma sample resulting in a hemoglobin concentration of 4 g/l.

To evaluate the possible impact of lipemia on results, 5 μl of Intralipid was added to 120 μl of non-spiked lithium heparin plasma sample so that a concentration of soya bean oil of 8 g/l was obtained. Analysis was performed in triplicates in random order. The spiked samples were investigated in comparison to lithium heparin plasma aliquots spiked with equal volumes of either 100 mM NaOH (in case of bilirubin), 0.09% NaCl (hemoglobin) or pure double-distilled water (in case of Intralipid).

#### Method comparison

In the method comparison study, the POCT was compared to a canine CRP assay run on two bench top analyzers (ABX Pentra 400; AU 5800). First, routine biochemical analysis including measurement of CRP was performed with the ABX Pentra 400 clinical chemistry analyzer of samples submitted to the Department of Veterinary Clinical Sciences, Clinical Pathology and Clinical Pathophysiology, Justus-Liebig-University Giessen, Germany. Specimens were included in the study if it was expected that sufficient residual sample volume was available to be re-analyzed with the POCT and the two bench top analyzers ABX Pentra 400 and AU 5800, respectively. The remainder lithium heparin plasma was then divided in three aliquots and stored at − 80 °C until analysis with the POCT and on the two bench top analyzers. Sampling was performed until 15 specimens for each CRP concentration range (low, moderate, high) were collected.

After finishing the period of sample acquisition and collecting a total number of 45 lithium heparin plasma specimens [31], two aliquots each were thawed at room temperature and re-analyzed in the Department of Veterinary Clinical Sciences, Clinical Pathology and Clinical Pathophysiology with the POCT and ABX Pentra 400 analyzer. The corresponding third aliquot of each sample was sent frozen to the commercial veterinary laboratory SYNLAB Vet GmbH, Augsburg, Germany to be analyzed on the AU 5800 analyzer. On both bench top analyzers, the same commercially available immunoturbidimetric canine specific CRP test was performed.

For all three analyzers, measurements were performed in duplicates to allow a direct comparison between the analyzers by calculation of the total observed error (TE_obs_) from intra-assay CV and %bias [31].

#### Establishment of reference intervals

For the reference interval study, lithium heparin plasma samples of 60 healthy adult dogs (> 1 year) were included. The dogs were presented at the Clinic for Small Animals, Faculty of Veterinary Medicine, Justus-Liebig-University, Giessen, Germany, as blood donors or for routine radiologic examination to screen for hereditary hip or elbow dysplasia. The dogs were classified as healthy and included in the study based on an unremarkable anamnesis and clinical examination.

### Statistical analysis

Statistical software programs (MedCalc, software version 16.2.1; Ostend, Belgium and GraphPad Prism 6 Software, GraphPad Software, Inc., La Jolla, USA) were used for statistical assessment of the obtained data.

#### Method validation

Results for CV%, bias% and TE_obs_ were compared to quality specifications derived from biologic variation published previously in the Total allowable error (TE) guidelines of the American Society of Veterinary Clinical Pathology (ASVCP, Table 1) [32]. For the purpose of the current study, numbers are rounded to one decimal place. Routine descriptive statistics were applied to calculate arithmetic means, standard deviations (SD), and CV. Normality was assessed using the Shapiro-Wilk Test.

**Table 1:** Quality specifications

Acceptance limits	Quality parameters	CV (%)	Bias (%)	TE (%)
- minimally acceptable		18.2	14.3	44.4
- desired		12.2	9.5	29.6
- optimal		6.1	4.8	14.8

Quality specifications are derived from biological variation as published in the addendum of the Total allowable error guidelines of the American Society of Veterinary Clinical Pathology [32]

*Abbreviations*: *CV* coefficient of variation, *TE* total allowable error

##### Linearity and recovery

Linearity under dilution was investigated by visual inspection of the correlation of observed CRP values plotted against a calculated (expected) CRP concentration. The difference between actual and theoretical CRP concentration was used to assess recovery after dilution:

$$ \mathrm{Recovery}\%=\frac{\mathrm{measured}\ \mathrm{concentration}}{\mathrm{expected}\ \mathrm{concentration}}\ast 100 $$

The quality goal for recovery after dilution was set at the range of 80–120% as recommended previously for validation of immunoassays [33, 34]. Linear and Deming regression analysis were performed to assess the correlation between expected and measured results.

##### Precision and lower limit of quantification

Quality requirements were fulfilled if the observed CV was < than the desired CV (CV_des_) for CRP (CV_des_, 12.2%) or at least < than the minimally acceptable CV (CV_min_, 18.2%) as reported previously [32].

Imprecision was computed based on mean and standard deviation (SD):

$$ \mathrm{CV}\%=\frac{\mathrm{SD}}{\mathrm{Mean}}\ast 100 $$

##### Interferences

The impact of possible interferences was evaluated by comparing control samples “spiked” with equal volumes of NaOH (instead of bilirubin), 0.09% NaCl (instead of hemoglobin) or pure double-distilled water (instead of Intralipid) as well as samples spiked with the interfering substances (bilirubin, hemoglobin, Intralipid) which were analyzed in triplicates.

The observed interference effect (d_obs_) was determined as the %bias between the mean of the test and the control samples:

$$ {d}_{obs}\%=\frac{mean_{test}-{mean}_{control}}{mean_{control}}\ast 100 $$

Bias between control and test sample (i.e., d_obs_ %) was considered acceptable according to the current literature if it was below the allowable TE, i.e. 29.6% (TE_des_) or even below 14.8% (TE_opt_) for canine CRP [32, 35].

#### Method comparison

Statistical analysis included the calculation of CVs from duplicate measurements for all three analyzers and concentration ranges. CV quality criteria were fulfilled if CV < CV_des_ (12.2%) or at least CV < CV_min_ (18.2%) as shown in Table 1 [32]. The means of the duplicate measurements were used for all further statistical tests.

For method comparison, a Bland Altman analysis was performed and both, %bias and absolute bias were calculated. Moreover, Passing Bablok analysis and calculation of Spearman’s rank correlation coefficient were performed. Correlations were considered “excellent” for Spearman’s rho (r_s_) =0.93–0.99, “good” for r_s_ = 0.80–0.92, “fair” for r_s_ = 0.59–0.79, and “poor” for r_s_ < 0.59, respectively [36].

As demonstrated in Table 1, quality criteria for bias were fulfilled if the mean observed %Bias_obs_ < %Bias_des_ (desired bias) or at least < %Bias_min_ (minimally acceptable bias) [32].

A Shapiro Wilk test was used to verify the assumption of normality. As non-normal distribution was present, a Kruskal-Wallis test was performed to assess a potential difference between median CRP measurements obtained for each analyzer.

To more objectively judge results, TE_obs_ was calculated and compared with quality specifications published previously for the measurements of CRP, i.e. the desirable total allowable error (TE_des_) and the minimally acceptable total error (TE_min_) [32].

TE_obs_ was calculated as published previously [27]: TE_obs_ = 2*CV% + bias%.

Quality requirements are fulfilled if TE_obs_ < TE_des_ (29.6%) or TE_min_ (44.4%) (Table 1) [32].

#### Establishment of reference intervals

As healthy dogs showed CRP values frequently (54/60 dogs) below the reported CRP concentration of the point-of-care analyzer (< 5 mg/l), a calculation of a definite reference interval was not possible.

## Results

### Method validation

#### Ease of use

The investigated point-of-care analyzer demonstrated to be a user-friendly in-house analyzer which could be easily used after a short training period.

Dilution buffer and test stripes have to be brought up from fridge to room temperature. Meanwhile, the analyzer is booting up and the clinician can use the time to centrifuge the blood sample to gain plasma (or serum). Then, 10 μl of the sample is pipetted in the dilution bottle filled with an adequate amount of dilution buffer and mixed 10 times. Afterwards, 100 μl of the dilution are applied on the test strip which is inserted in the analyzer. Measurement starts automatically and a result is provided after 5–10 min of kinetic measurement. Only if a new box of test stripes is opened, a calibration stripe for this batch of test stripes has to be inserted before measurement of a sample. All equipment (dilution bottle, test stripe) is for single use. One measurement including all preparations and waiting time for the reagents to acclimatize takes about 20–25 min. Expiration period of the test kit with 30 strips is 6 months at fridge temperature.

#### Linearity and recovery

**Fig. 1:**
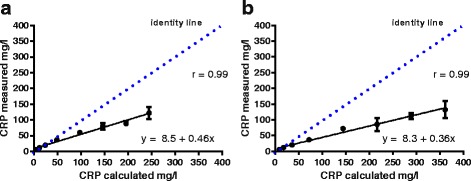
Linearity of two diluted lithium heparin plasma samples with high CRP concentrations. **a** Linearity under dilution for measurement of a canine serum sample originally containing 244.8 mg/l CRP as assessed with the Pentra 400 bench top analyzer. **b** Linearity under dilution for measurement of a canine serum sample originally containing 361.3 mg/l CRP as assessed with the Pentra 400 bench top analyzer. A serial dilution was performed in both cases to achieve 8 different CRP concentrations, i.e, 1.0, 0.8, 0.6, 0.4, 0.2, 0.1, 0.05, 0.025 parts of the original concentration

The results of the two linearity experiments are shown in Fig. 1 a) and b) as well as in Table 2 a) and b).

**Table 2:** Linearity and recovery

Dilution Factor	Expected concentration [mg/l]	Mean measured concentration [mg/l]	Recovery [%]	Bias_obs_ [%]	%Bias_obs_ < TE_des_ (29.6%)	%Bias_obs_ < TE_min_ (44.4%)
A						
0.025	6.1	6.5	106.8	6.8	Yes	Yes
0.05	12.2	11.9	97.0	3.1	Yes	Yes
0.1	24.5	19.8	81.0	19.0	Yes	Yes
0.2	49.0	36.5	**74.6**	25.5	Yes	Yes
0.4	97.9	59.8	**61.0**	**39.0**	**No**	Yes
0.6	146.9	79.8	**54.3**	**45.7**	**No**	**No**
0.8	195.8	88.0	**44.9**	**55.1**	**No**	**No**
1	244.8	122.3	**50.0**	**50.1**	**No**	**No**
B						
0.025	9.0	6.2	**68.7**	**31.3**	**No**	**No**
0.05	18.1	12.3	**68.3**	**31.7**	**No**	**No**
0.1	36.1	20.4	**56.6**	**43.4**	**No**	**No**
0.2	72.2	36.7	**50.8**	**49.2**	**No**	**No**
0.4	144.4	72.5	**50.2**	**49.8**	**No**	**No**
0.6	216.6	85.8	**39.6**	**60.4**	**No**	**No**
0.8	288.8	112.6	**39.0**	**61.0**	**No**	**No**
1	361.3	132.4	**36.7**	**63.4**	**No**	**No**

Linearity and recovery rates of CRP measurements performed with a canine serum sample containing A) 244.8 mg/l or B) 361.3 mg/l CRP respectively as determined on the ABX Pentra 400 analyzer

Quality parameters exceeding the quality criteria reported previously [32, 36], are marked in bold letters

*Abbreviations*: *Bias*_*obs*_ observed bias, *TE* total allowable error, *TE*_*des*_ desirable TE, *TE*_*min*_ minimally acceptable TE

As displayed in Fig. 1 a) and b), there was an excellent correlation between expected and measured results. For the POCT, linearity was given up to the highest CRP concentrations assessed of a mean of 123 and 130 mg/l.

Comparison between expected and measured results revealed a marked proportional Bias_obs_ ranging between 6.8 and 63.4% (Table 2). The Bias_obs_ and subsequently the TE_obs_ were exceeding quality requirements for samples with a dilution factor ≥ 0.025 and 0.1 respectively.

#### Precision and lower limit of quantification

Inter- and intra- assay CVs calculated from replicate measurements are shown in Table 3. As seen in the table, intra- assay CV was ≤8% in most cases and inter-assay CV ≤ 11%, respectively and thus < CV_des_ of 12.2%. The only exception were samples with a relatively low CRP concentration < 25 mg/L, where higher intra- and inter-assay CVs of 13.2–21.8% and 27.2% were obtained.

**Table 3:** Intra- and inter-assay CVs obtained from replicate CRP measurements with the POCT

Three CRP concentration ranges / analyzer	Dog^a^	Intra-assay CV (*n* = 10 replicates)			Inter-assay CV (*n* = 7 replicates)		
		Mean (mg/l)	SD (mg/l)	CV (%)	Mean (mg/l)	SD (mg/l)	CV (%)
≥ 10 - < 50 mg/l	1	21.3	4.7	**21.8**	23.2	6.3	**27.2**
	2	43.1	2.3	5.4	32.3	2.0	6.2
	3	23.1	3.7	**16.3**	15.4	0.8	5.0
	4	29.9	4.0	**13.2**	32.2	2.8	8.8
≥ 50 - < 100 mg/l	5	55.7	3.0	5.5	71.0	7.6	10.7
	6	79.8	4.7	5.9	74.4	5.5	7.3
≥ 100 - < 200 mg/l	7	113.9	7.4	6.5	102.4	7.2	7.0
	8	113.9	9.1	8.0	110.5	11.1	10.0

^a^Samples from different dogs were used for assessment of intra- and inter- assay CV

CV > CV_des_ (i.e., 12.2%) and CV > CV_min_ (i.e., 18.2%) is shown in bold letters

*Abbreviations*: *SD* standard deviation, *CV* coefficient of variation, *CV*_*des*_ desirable CV, *CV*_*min*_ minimally acceptable CV

In the LoQ study (Table 4), the lowest sample with a CRP concentration of 0.8 mg/l was correctly reported as < 5 mg/l, therefore the calculation of a CV was not possible. The CVs of 20 replicate measurements of samples with CRP concentrations of 10 mg/l and 20 mg/l respectively fulfilled the quality goal of CV_obs_ < CV_des_ of 12.2% (6.2%, 11%). The LoQ was set at 10 mg/l as this was the lowest CRP concentration evaluated that achieved the quality criteria.

**Table 4:** Determination of the lower limit of quantification

Three low CRP concentrations	Dog	Intra-assay CV (*n* = 20 replicates)			
		Mean (mg/L)	SD (mg/L)	CV (%)	% CV < CV_des_ (12.2%)
< 1 mg/l	1	< 5*	n.d.	n.d.	n.d.
~ 10 mg/L	2	11.5	0.7	6.2	Yes
~ 20 mg/l	3	16.8	1.8	11.0	Yes

Intra-assay CVs for the CRP analysis obtained from replicate measurements at low CRP values with the POCT

*values < 5 mg/l are generally reported as “< 5 mg/l”, therefore no further calculations were possible

*Abbreviations*: *SD* standard deviation, *CV* coefficient of variation, *CV*_*des*_ desirable CV, *n.d*. not done

#### Interferences

No interference was detectable up to a concentration of 800 mg/l bilirubin, 4 g/l hemoglobin and 8 g/l soy bean oil (Table 5).

**Table 5:** Observed interference effects of bilirubin, hemoglobin, lipid on triplicate CRP measurement with the POCT

Interferent	Mean CRP_control_ [mg/l] ± SD	Mean CRP_test_ [mg/l] ± SD	Mean bias [mg/l]	%Bias_obs_	%Bias_obs_ < TE_des_ (29.6%)	%Bias_obs_ < TE_opt_ (14.8%)
Bilirubin 800 mg/l	37.6 ± 5.6	39.8 ± 0.4	2.2	5.8	Yes	Yes
Hemoglobin 4 g/l	29.5 ± 1.7	30.1 ± 4.1	0.6	2.0	Yes	Yes
Soy bean emulsion 8 g/l	35.1 ± 1.4	39.1 ± 0.8	4.0	11.3	Yes	Yes

Test samples (CRP_test_) spiked with the interfering substances were compared to control samples (CRP_control_) spiked with equal volume of the diluent used in the test sample. %Bias_obs_ (observed bias) for the interfering substance was acceptable if %Bias_obs_ < desired total allowable error (TE_des_) and excellent if %Bias_obs_ < optimal total allowable error (TE_opt_) [32]

Mean %Bias_obs_ between control and spiked test samples was < TE_opt_ and thus fulfilling quality requirements.

### Method comparison

Overall, 45 samples were included for the comparison between the POCT and the Gentian canine CRP immunoassay run on the ABX Pentra 400 analyzer. For the comparison between the POCT and AU 5800, 2/45 samples were excluded due to insufficient sample volume.

Intra-assay CVs obtained for duplicate CRP measurements for the three evaluated analyzers are shown in Table 6. As seen in the table, median CVs <  1% and 2% were obtained for the automated bench top analyzers ABX Pentra 400 and AU 5800 with a range of 0%–3.2% and 0.3–5.6% respectively.

**Table 6:** Results of method comparison: Intra-assay precision

Intra-assay CV median (range) at three CRP concentration ranges / analyzer	Point Reader™ V	ABX Pentra 400	AU 5800
≥ 10 - < 50 mg/l	3.53% (0.0–38.91%)	0.62% (0.0–3.23%)	0.76% (0.26–3.0%)
≥ 50 - < 100 mg/l	3.50% (0.16–7.39%)	0.43% (0.08–2.21%)	1.68% (0.49–5.60%)
≥ 100 - < 200 mg/l	5.62% (0.40–16.60%)	0,30% (0.0–0.60%)	1.50% (0.52–2.27%)

Intra-assay CVs for the CRP analysis obtained from duplicate measurements performed on two automated bench top analyzers (ABX Pentra, AU 5800) and the point-of-care analyzer

*Abbreviations*: *CV* coefficient of variation

For the POCT, median intra-assay CVs were < 6%, however, there was a broad range especially in the low concentration range (0% to 38.9%).

**Fig. 2:**
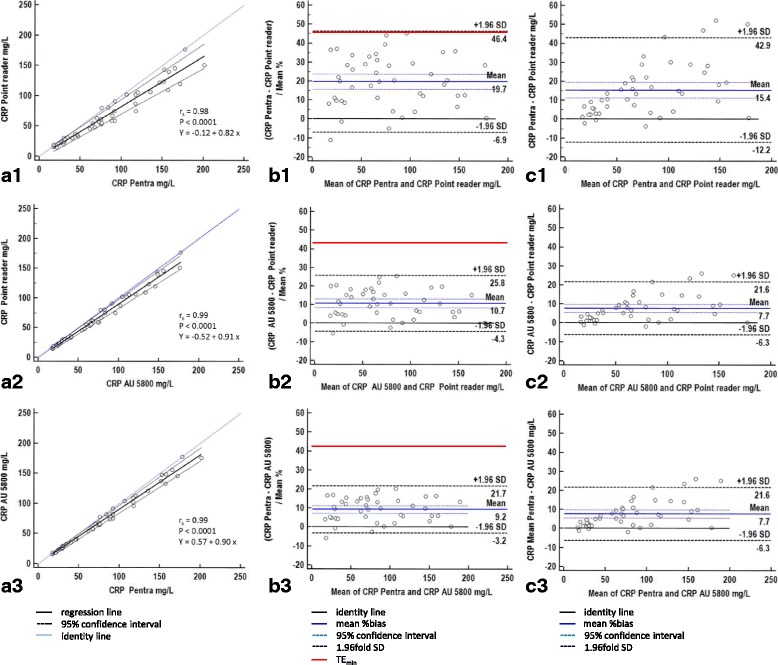
Results of method comparison between the POCT and two automated analyzers. The point-of-care analyzer was compared to a CRP assay run on two automated large bench top analyzers (ABX Pentra 400, AU 5800). A1–3: Passing- Bablok regression analysis with 95% confidence interval (CI) of CRP measurements performed with the three analyzers. A1: 95% CI of slope and intercept of the regression line were 0.74 to 0.90 and − 4.52 to 4.10, respectively. A2: 95% CI of slope and intercept were 0.86 to 0.95 and − 2.15 to 2.25, respectively. A3: There was a 95% CI of slope and intercept of 0.85 to 0.95 and − 2.75 to 2.02, respectively. B1–3: Bland-Altman difference plot demonstrating mean %Bias_obs_ with its 95% confidence interval and its 1.96fold standard deviation (SD) indicative of its limits of agreement. The minimally acceptable total allowable error (TE_min_) of 44.4% [32] represents the quality limit. C1–3: Bland-Altman difference plot demonstrating the absolute bias between CRP measurements obtained with three analyzers. For remainder of key, refer to Fig. 1b

Results of the method comparison study are shown in Fig. 2. There was an excellent correlation between CRP measurements obtained with the three analyzers (r_s_ ranging between 0.98 and 0.99, Fig. 2 A1–3).

Passing-Bablok regression analysis revealed a correlation of r_s_ = 0.98 between the data obtained with POCT and measurements of the ABX Pentra 400 with the regression eq. Y = − 0.12 + 0.82×. Regression analysis between POCT and AU 5800 revealed a Spearman’s rank correlation coefficient of r_s_ = 0.99 and the regression eq. Y = − 0.52 + 0.91×. In the additional correlation analysis between ABX Pentra 400 and AU 5800 using the same CRP assay, a Spearman’s rank correlation coefficient of r_s_ = 0.99 and the regression eq. Y = 0.57 + 0.90× was obtained.

**Fig. 3:**
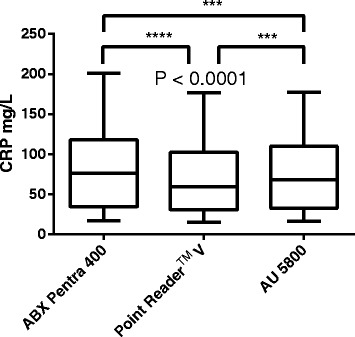
Results of method comparison: Differences of medians and ranges between the analyzers. Box- and - whisker diagram demonstrating median and range of the CRP measurements obtained with the three analyzers. The horizontal line in the boxes is consistent with the median, the whiskers indicate the range and the box represents the 25th -75th percentile

As demonstrated in Fig. 2 B1–2, Bland Altman analysis revealed a proportional Bias_obs_ of 10.7% and 19.7% between CRP measurement obtained with the POCT and the two automated bench top analyzers AU 5800 and ABX Pentra 400, respectively. All three analyzers revealed a growing absolute bias with increasing CRP concentration (Fig. 2 C1–3). Especially the POCT and ABX Pentra 400 analyzer disagreed considerably (Fig. 2 B1–2, C1–2) exceeding the %Bias_min_ of 14.3%. Bias_obs_ was lower between the POCT and the AU 5800 analyzer being < %Bias_min._ Interestingly, even between both bench top analyzers using the same CRP assay, a mean Bias_obs_ of 9.2% was seen (Fig. 2 B3), which, however, was still < both %Bias_des_ and %Bias_min_ of 9.5% and 14.3%, respectively. As shown in Fig. 3, there was a significant difference between mean CRP measurements obtained with all analyzers, whereby highest CRP measurements were obtained with the ABX Pentra 400 analyzer and lowest results with the POCT. Median (minimum to maximum) values obtained were 76.00 mg/l (17.05–201.4 mg/l), 59.35 mg/l (14.90–177.0 mg/l) and 68.18 mg/l (16.38–177.4 mg/l) for the ABX Pentra 400, the POCT and the AU 5800, respectively.

As shown in Table 7, quality requirements were fulfilled as TE_obs_ remained < TE_min_ of 44.4% for all comparisons. For the majority of comparisons, TE_obs_ was also < TE_des_ of 29.6%. The only exception was the comparison between the POCT and ABX Pentra 400 for CRP concentrations > 100 mg/l, where a TE_obs_ of 31.9% was obtained.

**Table 7:** Results of method comparison: Total observed error

TE_obs_ at three CRP concentration ranges / analyzer	ABX Pentra 400 vs POCT	AU 5800 vs POCT	ABX Pentra 400 vs AU 5800
≥ 10 - < 50 mg/l	27.3%	17.3%	10.7%
≥ 50 - < 100 mg/l	26.7%	16.7%	12.6%
≥ 100 - < 200 mg/l	**31.9%**	21.9%	12.2%

Total observed error (TE_obs_) for the CRP analysis performed on two automated bench top analyzers (ABX Pentra 400, AU 5800) and the point-of-care analyzer TE_obs_ > TE_des_ (i.e., 29.6%) but < TE_min_ (i.e., 44.4%) is shown in bold letters

*Abbreviations*: vs versus, *TE*_*des*_ desirable total allowable error, *TE*_*min*_ minimally acceptable total error

### Establishment of reference intervals

Overall, 60 clinically healthy dogs with a median age of 2 years (range 1–9 years) were included to evaluate a reference range for CRP as assessed with the POCT. Sex was equally distributed with 30 male (25 intact, 5 neutered) and 30 female (28 intact, 2 spayed) dogs. The breeds were represented as follows: 11 German Shepherd Dogs, 9 Golden Retrievers, 9 Labrador Retrievers, 7 mixed-breed dogs, 6 Bearded Collies, 3 Great Danes, 2 Australian Kelpies, 2 Dogues de Bordeaux, 2 Rough Collies, 2 Rhodesian Ridgebacks, 1 Bernese Mountain Dog, and 1 Border Collie, Boxer, Bullmastiff, Cane Corso Italiano, Hovawart, Magyar Viszla each.

Healthy dogs presented with CRP values ≤11.9 mg/l.

## Discussion

The POCT was capable to detect canine CRP with an excellent correlation with the automated immunoturbidimetric test run on large bench top analyzers in both, a university veterinary laboratory and a commercial veterinary laboratory, even though a proportional bias of the assay has to be considered. Generally, quality requirements were fulfilled as TE_obs_ remained < TE_min_ of 44.4% and for the majority of comparisons < TE_des_ of 29.6% [32]. This is in accordance with other POCTs for canine CRP [27]. Intra- and inter-assay CVs ranging from 0 to 24% have been reported for different other POCTs at low CRP concentrations which was generally consistent with the results obtained here [27].

For pathologically relevant CRP concentrations of > 50 mg/l, the POCT fulfilled quality requirements, i.e intra- and inter-assay CVs < CV_opt_ or at least < CV_des_. Other than expected and described previously for various assays [28, 29], highest CVs were not obtained for samples in the lowest CRP concentration range, but at low to medium CRP concentrations of 20–30 mg/l. Possible explanations may be “hardware errors” due to biochemical background reactions of the assay itself or an impairment of the optical detection. Variations between different lots or individual test stripes of the assay are another possible source of error. However, in case of lot-associated variations, outliers would be observed at several different CRP concentration ranges rather than at one single concentration range. Alternative explanations are user-dependent errors as dilution- and pipetting errors. User-dependent errors, however, were minimized as all measurements were performed by two trained persons. Although analytical imprecision in the concentration range between CRP concentrations of 20 and 30 mg/l is not desirable, it is considered of minor clinical relevance as it generally does not have an impact of the clinical decision, i.e. interpretation of the CRP results as mildly increased. However, the possibility of a CV of 20–30% has to be taken into account when interpreting follow-up examinations of the patient.

The current evaluation clearly showed that a bias between the methods has to be expected even when the same test is used as it was the case for the ABX Pentra 400 and the AU 5800 bench top analyzer. The current results show that the bias is not only caused by the different ability of the antibody used in the test to bind canine CRP but also by the analyzer itself. The impact of the analyzer on assay results has already been discussed in the literature for the same CRP assay which is used as reference method in the current study [27, 29]. Moreover, an impact of different lots of the Gentian Canine CRP Immunoassay on the results cannot be entirely ruled out.

As demonstrated here, the bias and thus also the TE_obs_ are highly dependent on the reference method (i.e., the combination of assay and analyzer) used for comparison. While %Bias_obs_ between the POCT and the bench top analyzer AU 5800 was at least fulfilling %Bias_min_ [32], %Bias_obs_ was slightly higher than Bias_min_ in the comparison between the POCT and the other bench top analyzer, ABX Penta 400. Other than expected, even the comparison between both bench top analyzers using the same assay revealed a mean %Bias_obs_ > than optimal bias %Bias_opt_. The results obtained here, demonstrate clearly the impact of the choice of the reference method on the results.

Unfortunately, a true gold standard is not available for the measurement of canine CRP, which is a major limitation of all method validation studies. Considering the %Bias_obs_ between both large bench top analyzers evaluated here and using the same assay, a Bias_obs_ of ~ 10% between the POCT and the AU 5800 can be considered as good while a Bias_obs_ of ~ 20% between the POCT and the ABX Pentra 400 may at least be considered as satisfying. Regarding the literature, a mean %bias ranging between ~ 10–30% is frequently reported in method validation studies for POCT designed for canine CRP analysis [27]. Due to the Bias_obs_, it can be concluded that test- and analyzer -specific reference intervals and clinical decision limits have to be established. Follow-up examinations should be therefore performed with the same method and analyzer [27]. Bias between results obtained with different analyzers should be also kept in mind, when classifying the severity of CRP increase as mild, moderate or severe. Also, CRP clinical decision limits reported in literature may not be applicable for the POCT [9]. As the POCT correlates well with the reference method (*r* > 0.975) [31], the regression equation may be used to adapt values reported by the POCT to values of the Gentian Canine CRP Immunoassay [37], i.e. a CRP value of 100 mg/l on the ABX Pentra 400 is consistent with 80 mg/l on the POCT and 90 mg/l on the AU 5800, while a value of 50 mg/l is consistent with 40 mg/l (Y = − 0.12 + 0.82 x) and 45 mg/l (Y = − 0.52 + 0.91 x), respectively. Still, the option to use the equation of the regression analysis has to be considered carefully and is not recommended for general use (i.e, incorporation into analyzers´ software) as measuring errors potentiate via mathematical adaption and the true origin of results becomes non-transparent.

The linearity experiment demonstrated linearity of the POCT up to a CRP concentration of 130 mg/l as well as an excellent correlation between measured and calculated values. However, the slope of the regression line was markedly below 1 also reflecting a marked proportional bias.

The evaluation of the lower limit of quantification was impaired by two facts: First, the POCT has an internal technically predetermined “limit of quantified report”. Low CRP concentrations < 5 mg/l are generally reported as “< 5 mg/l” by the analyzer. Second, results of repeated measurements of the analyzer at low CRP concentrations < 30 mg/l, CVs are good to excellent in most cases, but single “outliers” occurred resulting in a high CV (Tables 3 and 4). Therefore, it is questionable if the evaluation of two samples with low CRP concentration levels above the reported limit of the analyzer is enough to determine a lower limit of quantification. A higher number of analyses of samples with CRP values > 5 mg/l but < 30 mg/l would be needed to increase the validity of the set limit. Regarding our own observations (Table 4), the limit of 5 mg/l fixed internally in the analyzer appears to be a good limit for reported values.

No interferences in clinically relevant ranges of up to 800 mg/l bilirubin, 4 g/l hemoglobin and 8 g/l soy bean oil (Intralipid) could be detected. As metabolic disorders associated with icteric, hemolytic or lipemic samples occur frequently in patients with inflammatory diseases [20] or emergency patients, the lack of impairment by these circumstances is considered an advantage. In contrast, interferences have been reported previously for other CRP assays, although they had been considered without clinical relevance [38].

In our study, statistical computation of an exact reference interval for healthy dogs was not possible as in the majority of dogs CRP values < 5 mg/l were observed. Still, this is not a disadvantage as very low CRP values are not of clinical relevance in canine medicine. The data demonstrate healthy dogs to show CRP values < 12 mg/l. This is comparable with most reported reference intervals for canine CRP established for various immunoassays in literature [16, 18–20, 39] or our own unpublished data for the Gentian Canine CRP Immunoassay on the ABX Pentra 400 with a reference interval ≤ 10.8 mg/l CRP.

Nevertheless, another recent study [40] also reported a wide range of normal CRP results (0.07–24.7 mg/l) obtained in 76 healthy dogs using a human-based assay (High linearity CRP, Randox, Crumlin, United Kingdom). In the previous study, the clinical decision limit to differentiate between healthy dogs or dogs without systemic inflammation and dogs with several types of diseases associated with systemic inflammation was set at a CRP concentration of 16.8 mg/l based on receiver operating characteristic analysis. In contrast, in another study evaluating a semiquantitative near-patient CRP assay, a higher CRP concentration of 35 mg/l is used as a cut-off value to discriminate “positive” and “negative” CRP samples [24]. However, given the bias even between analyzers using the same assay, potential bias as well as the CV at this concentration level have to be considered. In case of marked bias, a laboratory-specific cut-off value has to be defined. Moreover, especially if a relatively high CV is present as observed for the POCT at this concentration range, a grey zone rather than a definite cut-off value should be used which can be calculated based on the analytical CV and biological variation, i.e. within dog variation as described previously [41].

Limitations of the study included the fact that quality specifications for CV, bias and TE based on biological variation as published in the total allowable error guidelines of the ASVCP [42] were applied here, however, they are judged as too stringent for method validation studies [42] and are even stricter than quality goals in human medicine [43]. As no adequate alternative is available for veterinary medicine, they have been used also in previous studies evaluating canine CRP assays [29].

Moreover, a possible prozone effect was not investigated in our study, however, there is no evidence of a prozone effect for a CRP range ≤ 360 mg/l (equaling 130 mg/l as assessed with the POCT) which covers a large part but not the total range of possible CRP results observed in diseased dogs [40, 44].

## Conclusion

Overall, the species-specific Point Reader™ V is an easy-to-use POCT suitable to detect canine CRP in lithium heparin plasma samples precisely in concentrations being relevant for clinical decision making. The POCT correlates well with an already established immunoturbidimetric species-specific CRP assay run on large bench top analyzers, however, the bias with other methods and analyzers has to be considered. Thus, CRP results assessed by different assays and/or different analyzers are not directly comparable in follow-up examinations.

## Abbreviations

APP: acute phase protein(s)
Bias_des_: Desired bias
Bias_min_: Minimally acceptable bias
Bias_obs_: Observed bias
Bias_opt_: Optimal bias
CRP: C-reactive protein
CV: Coefficient of variation
CV_des_: Desired coefficient of variation
CV_opt_: Optimal coefficient of variation
ELISA: Enzyme-linked immunosorbent assay
LoQ: Limit of quantification
POCT: Point-of-care test
SD: Standard deviation
TE: Total error
TE_des_: Desired total allowable error
TE_min_: Minimally acceptable total allowable error
TE_obs_: Observed total allowable error
TE_opt_: Optimal total allowable error
